# Non-Watson–Crick RNA synthesis suited to origin functions

**DOI:** 10.1261/rna.063974.117

**Published:** 2018-01

**Authors:** Deepa Puthenvedu, Irene Majerfeld, Michael Yarus

**Affiliations:** Department of Molecular, Cellular and Developmental Biology, University of Colorado, Boulder, Boulder, Colorado 80309-0347, USA

**Keywords:** AppA, GppG, gene, coenzyme, pA, pG

## Abstract

A templated RNA synthesis is characterized in which G^5′^pp^5′^G accelerates synthesis of A^5′^pp^5′^A from pA and chemically activated ImpA precursors. Similar acceleration is not observable in the presence of UppU, CppC, AppG, AppA, or pG alone. Thus, it seems likely that AppA is templated by GppG via a form or forms of G:A base-pairing. AppA also appears, more slowly, via a previously known untemplated second-order chemical route. Such AppA synthesis requires only ordinary near-neutral solutions containing monovalent and divalent salts, and rates are only slightly sensitive to variation in pH. Templated synthesis rates are first order in pA, ImpA, and template GppG; thus third order overall. Therefore, this reaction resembles cross-templating of AppA on poly(U), but is notably slower and less sensitive to temperature. Viewing AppA as a coenzyme analog, GppG templating provides a simpler molecular route, termed para-templating, to encoded chemical functions. Para-templating can also arise from a single, localized nucleobase geosynthetic event which yields purines. It requires only a single backbone-forming chemistry. Thus it may have appeared earlier and served as evolutionary precursor for more complex forms of encoded genetic expression.

## INTRODUCTION

### Coenzyme-RNAs

Extensive experimental evidence supports the hypothesis of coenzyme activity during and after an RNA world. Bona fide coenzymes can be synthesized at the 5′ terminus of RNA molecules via ribozyme activity (Huang et al. 2000), or assembled from nucleotides bound on a ribozyme surface (Huang et al. 1998). Moreover, coenzymes are readily incorporated into RNA. They can be added by ribozymes to RNAs carrying a specific sequence tag (Jadhav and Yarus 2002), or acquired at a 5′ terminal position by acting as initiators of transcription (Coleman and Huang 2005). From 5′-coenzyme-terminated sequence libraries, ribozymes that mimic the modern biosynthesis of acyl-CoA's can be selected (Coleman and Huang 2002; Jadhav and Yarus 2002). Ancient reliance on cofactor RNAs for oxidoreduction is further supported by selection of NAD-ribozymes that both oxidize and reduce a substrate (Tsukiji et al. 2003, 2004).

### Coenzyme-peptides

Modern protein enzymes still bear clear marks of RNA ancestry. AMP-containing coenzymes are particularly likely remnants of RNA enzymes (White 1976). Of more than 4000 enzymes currently listed in the Enzyme Commission database, 35% depend on an organic cofactor (leaving aside inorganic cofactors like metal ions; calculated from data in Fischer et al. 2010). However, this statistic still does not convey the importance of RNA cofactors—some types of enzymes, like oxidoreductases, rely on organic cofactors in >80% of modern cases (Fischer et al. 2010). Accordingly, this essential metabolic sector is mostly RNA-dependent even today.

### Free coenzymes

But coenzyme molecules are plausibly older than the RNA world, having potentially been conserved for gigayears (Yarus 2011). Redox reactions today are largely carried out by enzyme-bound ribonucleotides like NAD and FAD. Such molecules also perform numerous related oxidoreduction reactions when free in solution (Richter 2013). Thus, not only do universal cofactors persist to the present, arguing for descent from ancient dinucleotides, but dinucleotides containing adenine often catalyze peptide-independent solution reactions related to their cofactor roles. There is, accordingly, a cogent argument for free cofactor molecules with ancient redox functions, afterwards acting as RNA coenzymes, and subsequently found as cofactors to protein catalysts.

### Encoding coenzyme congeners

This notion is greatly strengthened because information for coenzyme-like molecules can be encoded in simple linear ribonucleotides, modeled by poly(U) or poly(C). These linear homopolymers encode coenzyme-like AppA or GppG, respectively, apparently by pyrimidine–purine base-pairing. For example:

pA+ImpA→3′−5′poly(U)5′−5′AppA+Im(Puthenvedu et al. 2015)

pG+ImpG→3′−5′poly(C)5′−5′GppG+Im(Majerfeld et al. 2016)

Linear polymer templates can be envisioned as products of clay-mediated polymerization (Ferris et al. 1996) of activated nucleotides. In this formulation, linear homopolymers from clay act as primitive “genes,” and coenzyme-like ribodimers act as “gene products.” Such products potentially support a phenotype because they contain at least one reactive nucleotide, so that an NppN* molecule more efficiently performs a chemical or physical transformation which can subsequently be selected. Supposing that related dinucleotides can be regarded as congeners of coenzymes like NAD (Yarus 2011), a chemical phenotype could have been inherited simply under primordial conditions (Yarus 2017).

This is not merely hypothetical. If millimolar concentrations of two complementary, partly activated nucleotides became available in the same place on an early Earth, selection for a favorable dinucleotide product would readily initiate inheritance of templated dinucleotide synthesis (Yarus 2017). Such evolution easily uses sporadic supplies of unstable nucleotide precursors, and happens rapidly. Details of the environment are important. Such selections are more rapid in recently established pools under continuous danger of termination, which strongly depend on a dinucleotide property to survive (Yarus 2017). Such successful evolutionary episodes are aided by intrinsic pool properties like nucleotide accumulation and chance utility (Yarus 2013, 2016), and rely on easily selected entropic catalysis intrinsic to template function (Yarus 2017).

Thus, we envision emergence of genetic behavior, by a route whose plausibility can be numerically demonstrated. We argue below that this narrative for appearance of inherited chemical proficiency can be further simplified. Both template and templated product can be 5′–5′ ribodinucleotides. That is, we now demonstrate a smaller, simpler reaction center which uses just one type of chemical reaction to create both model templates (GppG) and a model encoded coenzyme-like product (AppA).

## RESULTS

### Measurement of products

**FIGURE 1.**
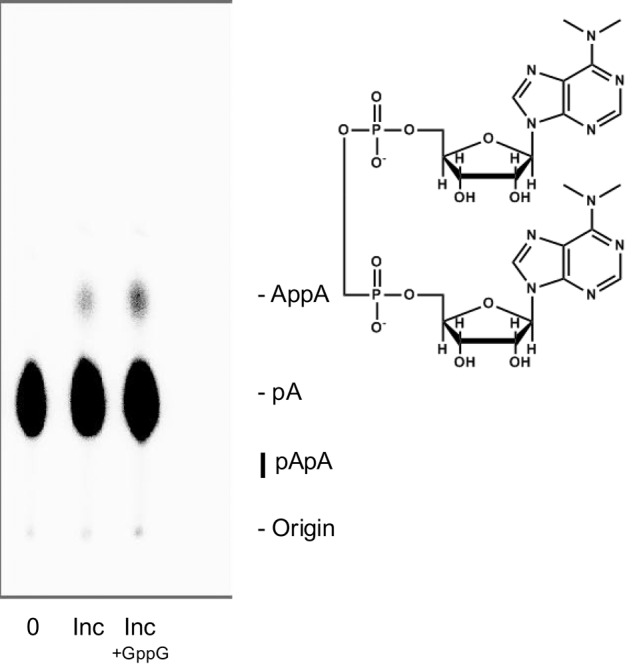
Polyethylene imine TLC resolves [^32^P] products. The structure of AppA is shown to the *right* of its label. The lane marked “0” is an unincubated complete reaction, “inc” is the untemplated reaction after 19 h at 12°C, and “inc + GppG” implies the same incubation but with 10 mM GppG added. This is the chromatographic system previously used for detection of AppA (Puthenvedu et al. 2015), and the identity of the product has been confirmed by HPLC (D Puthenvedu and M Yarus, in prep.). By determining the fraction of total radioactivity in the product spot, product concentration can be calculated from known total [pA]. The bar marked pApA at the *lower right* of the chromatogram shows the position of 2′–5′ pApA (*top* of the bar) and 3′–5′ pApA (*bottom*), if present.

Figure 1 shows TLC fractionation of typical reactions containing 5′[^32^P]A, along with activated pA (ImpA; 2-methyl-imidazolyl-adenylic acid [Joyce et al. 1984]) ± GppG. The single resolved product spot, ahead of substrate 5′[^32^P]A, is AppA, which appears spontaneously, but at an accelerated rate when GppG is added. Note that as for the substantially different cross-templating systems (Puthenvedu et al. 2015; Majerfeld et al. 2016) a single, stable, 5′–5′ dinucleotide product is prominent, both with and in the absence of the putative template. In particular, there is no observable 2′–5′ or 3′–5′ pApA synthesis. Increased synthesis with GppG added to the reaction containing 5′ [^32^P]A is clearly seen and quantitated by phosphorimaging. In these three kinds of unusual templating, therefore, accelerated 5′–5′ AppA synthesis is easily detected, but products with 2′–5′ or 3′–5′ polarities are almost absent.

**FIGURE 2.**
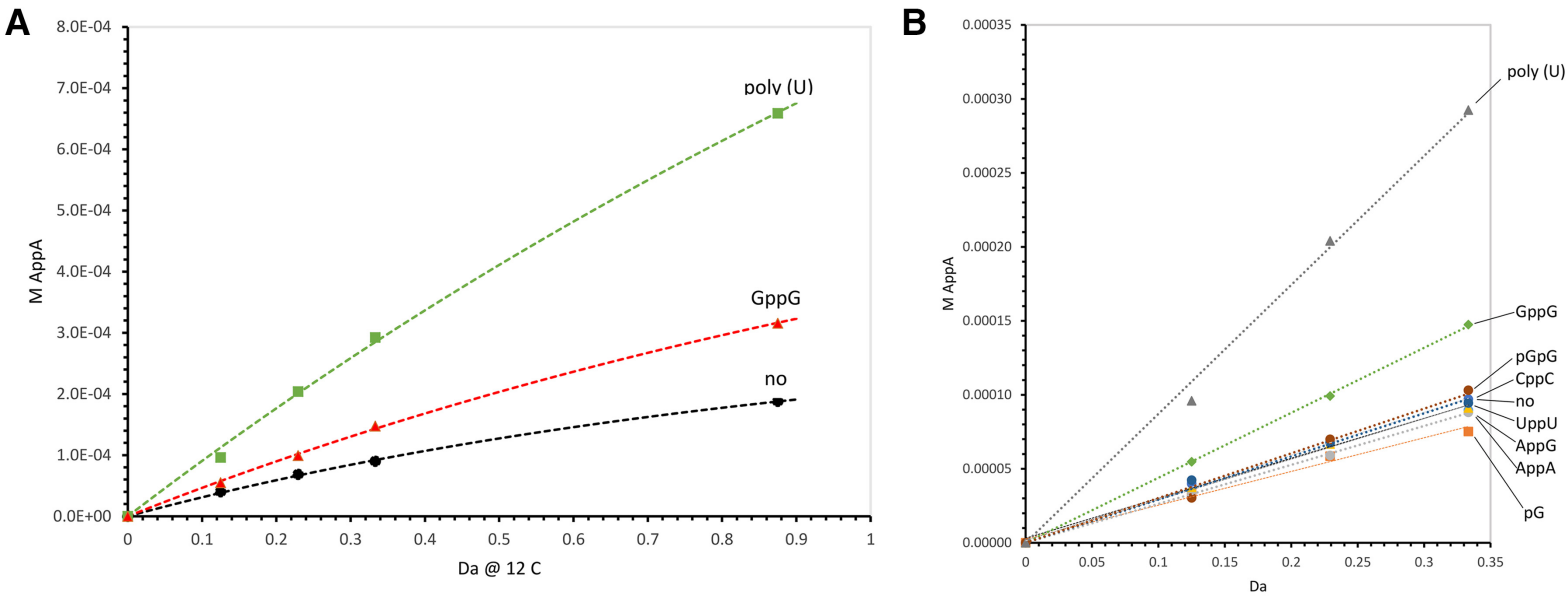
(*A*) Comparison between the cross-templating synthesis of AppA and the current GppG-templated reaction. [^32^P]AppA concentration is plotted versus time at 12°. With 10 mM poly(U)—green squares, with 10 mM GppG—red triangles. The curve marked “no” has only nucleotides, no template. Curved dashed lines are calculated least squares fits to experimental points (using the model in Materials and Methods). (*B*) Early kinetics of AppA synthesis. Reactions contain 10 mM pA, 10 mM 2meImpA, and a trace of [5′ ^32^P]pA. Least squares linearity of the plots suggests that they yield rates substantially unaffected by 2meImpA decay. Labels at the *right* resolve superposed data for 10 mM ribodinucleotides or nucleotide tested for template activity. (No) No template, chemical reaction alone. Triangles at the *top* are poly(U) data.

This chromatographic method can therefore be used to quantitate the velocity of AppA synthesis under varied conditions, as shown in Figure 2. For example, note that GppG is at a molecular concentration of 10 mM, and poly(U) is at a total nucleotide phosphate concentration of 10 mM. The result is that poly(U) is actually at the lower molecular concentration, but supports a higher rate of synthesis than GppG. The curvature of the plots embodies decay of the ImpA, which allows total rates of activated nucleotide disappearance to be determined. Materials and Methods contains kinetic details, which allow determination of kinetic orders and rate constants (Table 1).

**FIGURE 3.**
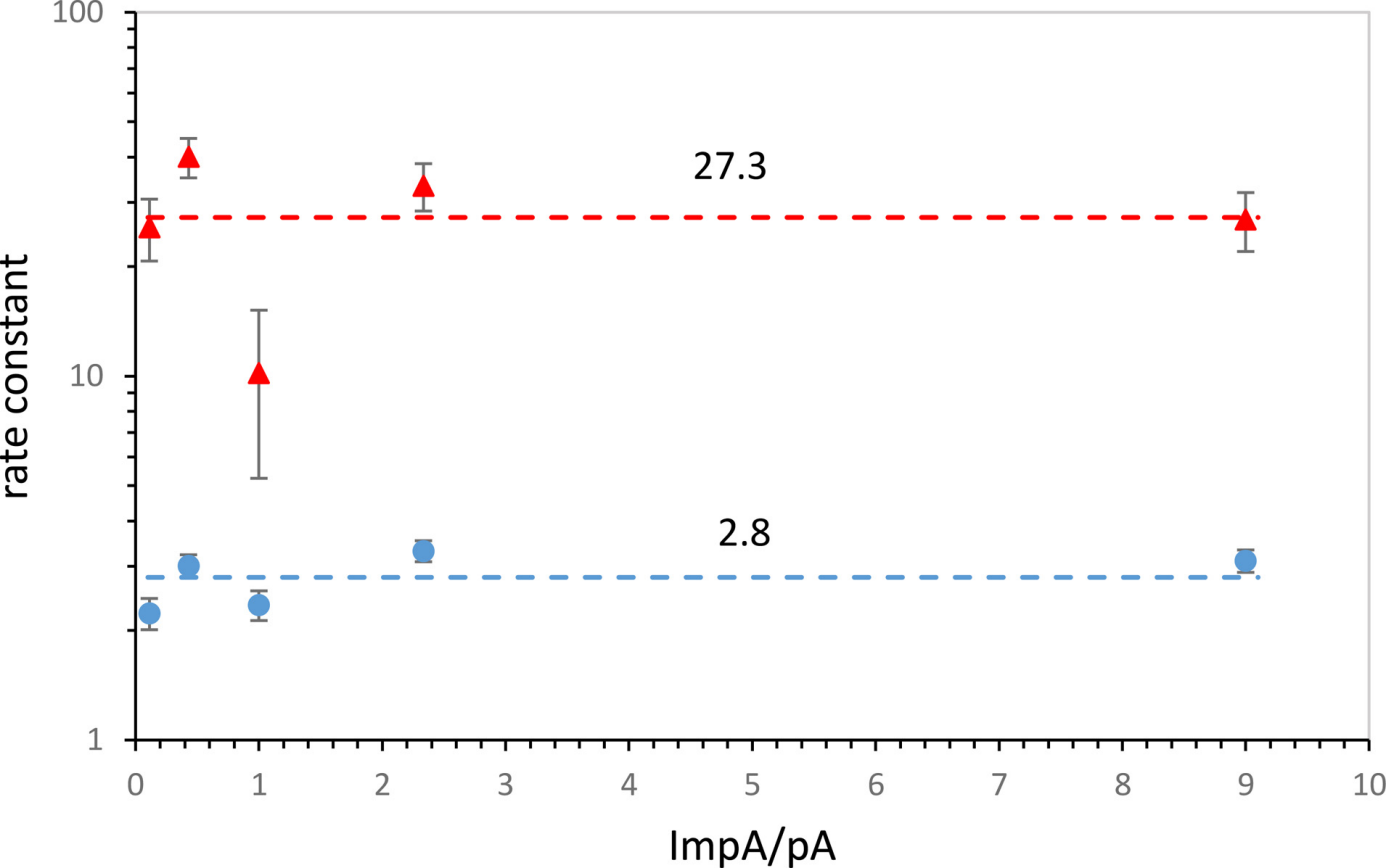
Rate constants and order of the reactions. Rate constants come from fitting 2-d reaction courses to the kinetic model in Materials and Methods. (*Top* red triangles) Templated reaction (rate constant M^−2^ Da^−1^); (*bottom* blue circles) chemical reaction (rate constant M^−1^ Da^−1^).

**TABLE 1.**
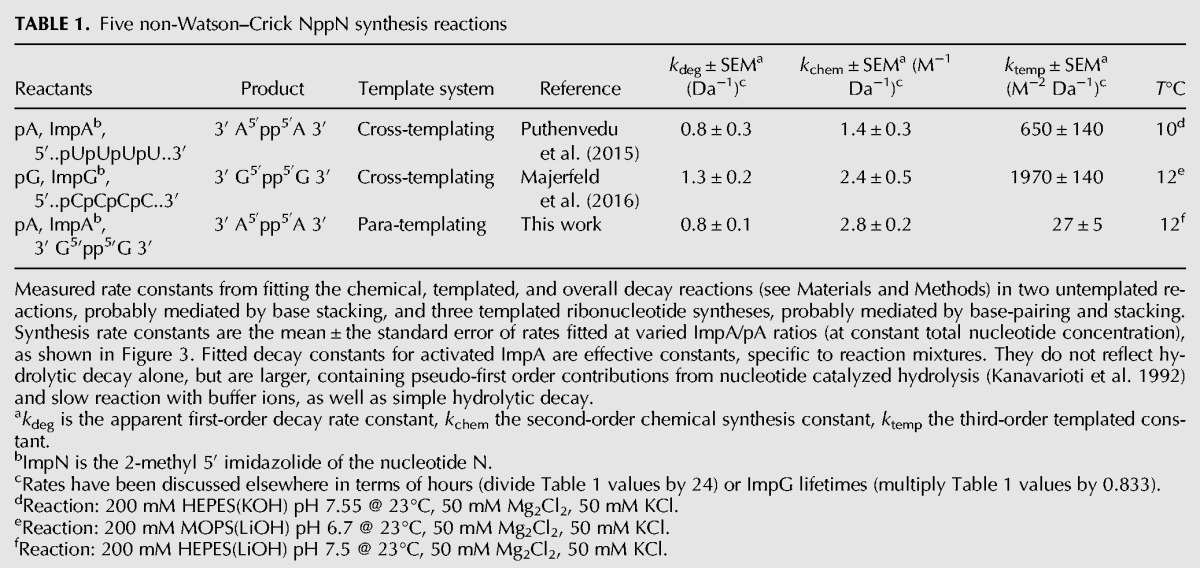
Five non-Watson–Crick NppN synthesis reactions

### Template specificity

Figure 2B contains initial velocities of reaction with several candidate templates and closely related molecules. Strikingly, related nucleotides do not stimulate as does GppG. Even 3′–5′ pGpG is almost indistinguishable from no addition. UppU, CppC, AppA, and AppG also have no significant effect. The 5′ mononucleotide pG alone is also unlike GppG, and appears slightly inhibitory. While poly(U) is stimulatory, UppU is inactive. Restriction of increased synthesis to GppG and poly(U) suggests that they alone bring pA and ImpA together in a reactive configuration. AppG and AppA in Figure 2B are particularly notable; they contain two purines, but are inactive. Thus, GppG is most likely acting via two A:G base pairs, somewhat as does poly(U) ordering two A's via U:A pairs (Puthenvedu et al. 2015; see Discussion).

### Reaction order for nucleotides

For quantitative purposes, the rates and rate equations (orders) of these syntheses are needed. Orders are determined by rate analysis using varied concentrations of reactants. For the nucleotides, total concentration is maintained constant to minimize bulk concentration effects, and the ratio of pA to ImpA is varied 90-fold. Observation of the same apparent rate constants for the chemical and templated reactions over this range means that both untemplated and templated synthesis are first order in both nucleotides because that assumption, within error, yields the same rate constant on fitting to data at highly varied nucleotide ratios.

### Reaction order for the putative template

**FIGURE 4.**
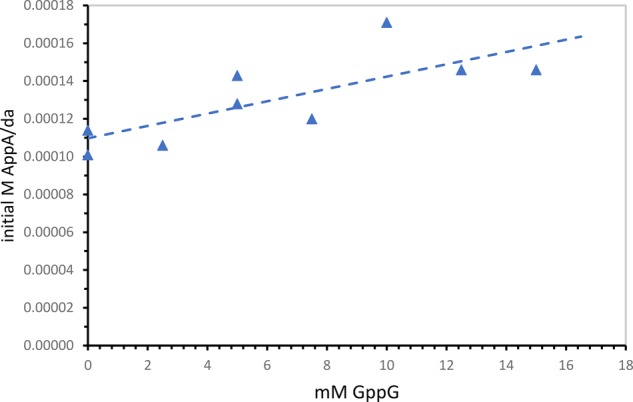
Initial AppA synthesis rates increase with GppG concentration. Here initial rates are determined using 10 mM nucleotides, with GppG varied up to 15 mM molecular concentrations. Thus the *y-*axis extrapolate defines the chemical reaction rate.

GppG can also be varied, and rates determined. AppA synthesis increases approximately linearly (Fig. 4), indicating that the templated reaction is first order in GppG. Thus the chemical reaction is second order overall in the nucleotides, and the GppG-stimulated reaction is third order overall: first order in two A nucleotides plus first order in GppG. This resembles previous cross-templated reactions, which behave in a similar second- and third-order fashion overall (Puthenvedu et al. 2015; Majerfeld et al. 2016).

### Comparison with cross-templating

Because all rate constants have the same orders, it is possible to compare velocities for different non-Watson–Crick templating examples. Most relevantly for quantitative treatment of these reactions, the present GppG reaction is notably slower. Cross-templated synthesis of GppG on poly(C) is of the same order, but about threefold faster than production of AppA on poly(U). However, para-templated production of AppA is 24-fold slower than production of the same product on poly(U), or 73-fold slower than synthesis of GppG on poly(C). This disparity is understandable, because both poly(U) and poly(C) templates are arguably better ordered, and therefore more effective in catalyzing the conjunction of nucleotides for reaction (Yarus 2017), than is GppG, which has no flanking nucleotides to help constrain its paired nucleotides. To discuss this concisely, we call the new reaction para-templating. Para- is a combining form that usually means “alongside” or “related to,” so it is appropriate for a reaction in which both template and product have no net polarity (they are symmetric; compare Fig. 1) and the same backbone.

### pH variation is small

**FIGURE 5.**
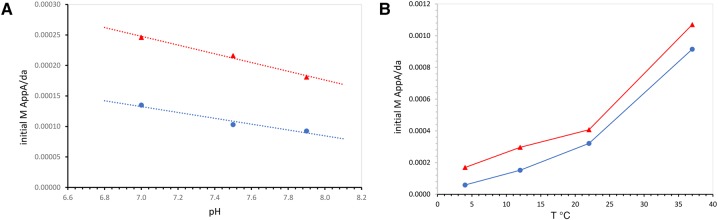
(*A*) Para-templating rates at varied reaction pH. Buffer pH is that measured during titration of HEPES with LiOH at 23°C. (Red triangles) Total rate, chemical plus templated at 12 mM GppG; (blue circles) chemical rate. (*B*) Initial reaction rate versus temperature, 4°C–37°C. (Red triangles) Total rate, with 12 mM GppG; (blue circles) chemical rate.

Other properties of the reaction give some hints about its nature. Neither untemplated nor templated rates are very dependent on pH; but both increase slightly under acid conditions (Fig. 5A). Thus the introduction of the more rapid GppG reaction does not significantly change the pH response of an untemplated chemical reaction. While cancellation of pH effects from widely spaced pK_A_’s is possible, the simplest interpretation of these data is that neither untemplated nor templated reaction involves an unusual tautomer (protonated or deprotonated form) of either pA, ImpA, or for that matter, GppG.

### Temperature variation

Reaction rates are very responsive to temperature (Fig. 5B). Notably, para-templating is observable throughout the 4°C to 37°C range studied. The untemplated (chemical, stacking) rate increases from 4°C through 37°C, and while the GppG-templated reaction is always present, it does not increase with temperature to the same extent. Thus para-templating is a smaller fraction of the total AppA synthesis reaction at higher reaction temperature (Fig. 5B), where the chemical reaction is predominant.

Nevertheless, persistence of para-templating at 37°C in Figure 5B is notable. Previous cross-templating reactions are more sensitive, acting as if they take place in a temperature-unstable reaction center. That is, cross-templated reactions cease suddenly at a lower temperature—by 20° C for AppA synthesis stimulated by poly(U) (Puthenvedu et al. 2015) and by 37°C for GppG synthesis on poly(C) (Majerfeld et al. 2016). Chemical synthesis increases regularly with temperature throughout, as do ordinary chemical reactions. It may be evolutionarily significant that GppG para-templating appears more resistant to temperature variation than are cross-templating reactions.

The persistence of para-templating to elevated temperature is thus not fully understood, but we tentatively take it as paralleling the discussion of Table 1 rate constants above. Para-templating takes place in a less-cooperatively formed reaction center whose temperature response is more akin to the chemical/stacking background reaction (compare Fig. 5B) than to the helical reaction center for cross-templating nucleotides (Puthenvedu et al. 2015).

## DISCUSSION

### Specificity of the para-templating reaction

GppG specifically stimulates the assembly of AppA from pA and an activated form of pA (Fig. 2B). Other NppN, including AppG, do not have this activity. Apparent templating is based on a novel backbone configuration; a symmetric purine template with two 3′ ends encodes a purine ribonucleotide product with the same structure.

pA+ImpA→5′−5′GppG⁡5′−5′AppA+Im

Though G–A base-pairing likely underlies these observations (Fig. 2B), a pairing model for the reaction center is still uncertain. AG base pairs in RNA structures are not only frequent (Traub and Sussman 1982), but highly varied in form. In a base pair database based on 9000 existing high-resolution RNA structures (http://ndbserver.rutgers.edu/ndbmodule/services/BPCatalog/bpCatalog.html), there are six frequent configurations for AG pairs with two H-bonds or more (based on the geometric classes of Leontis and Westhof 2001). Such recurring AG pairs engage three different faces of paired A, and two faces of paired G. The six abundant AG pairs contain only normal tautomers; unusual AG tautomeric pairs (Topal and Fresco 1976) being neglected here because of the limited sensitivity (Fig. 5A) of para-templating to incubation pH. Such evident base-pairing versatility for A and G is likely one reason why UppU is inactive but GppG is functional as an apparent template, though both can potentially pair with A.

In the little-explored structural environment of a paired 5′–5′ dinucleotide, the two base pairs of a para-templating reaction center might differ in structure. Accordingly, based on known high-resolution structures there are 21 initial paired structures to consider for a GppG/AppA product complex. Clarification of such variability might require solution of a number of high-resolution structures. However, such a large study would be of great interest, because these structures would rationalize the base specificity of para-templating. An interesting possibility is: If template-product pairs take more than one form, GppG-templated systems may have more than one product, making GppG an exceptionally versatile source of encoded information.

A specificity question of particular significance is whether AppA can, under overlapping conditions, catalyze a reciprocal reaction to encode GppG. Existence of the AppA-templated reaction would define a system capable of indefinite para-templated replication, which relies on transiently base-paired, but nonstandard dinucleotides, existing mostly as unpaired molecules (Yarus 2012). This would be of immediate evolutionary interest (Yarus 2012, 2017).

### All nucleobases for para-templating from one primordial synthesis

This para-templating reaction potentially simplifies early evolution by requiring only two interacting ribonucleotides, both purines. Francis Crick (Crick 1968) (following a suggestion from Leslie Orgel) suggested primordial usage of purines only, allowing pyrimidines to appear later. Chemical models of early geosynthesis from NH_4_CN (Oro and Kimball 1961) yield both A and G nucleobases (Levy et al. 1999), making a purine-only origin chemically plausible. Though guanine is less abundantly made, it can occur in the same order of concentration as adenine (Borquez et al. 2005). Other possible nucleobase sources such as impact on a reducing atmosphere also supply the synthetic impetus for both standard purines (Ferus et al. 2017). Most relevantly, adenine and guanine of extraterrestrial origin occur together in multiple carbonaceous chondrite meteorites (Callahan et al. 2011).

### Candidates for A*, A, and G emerge together

**SCHEME 1.**
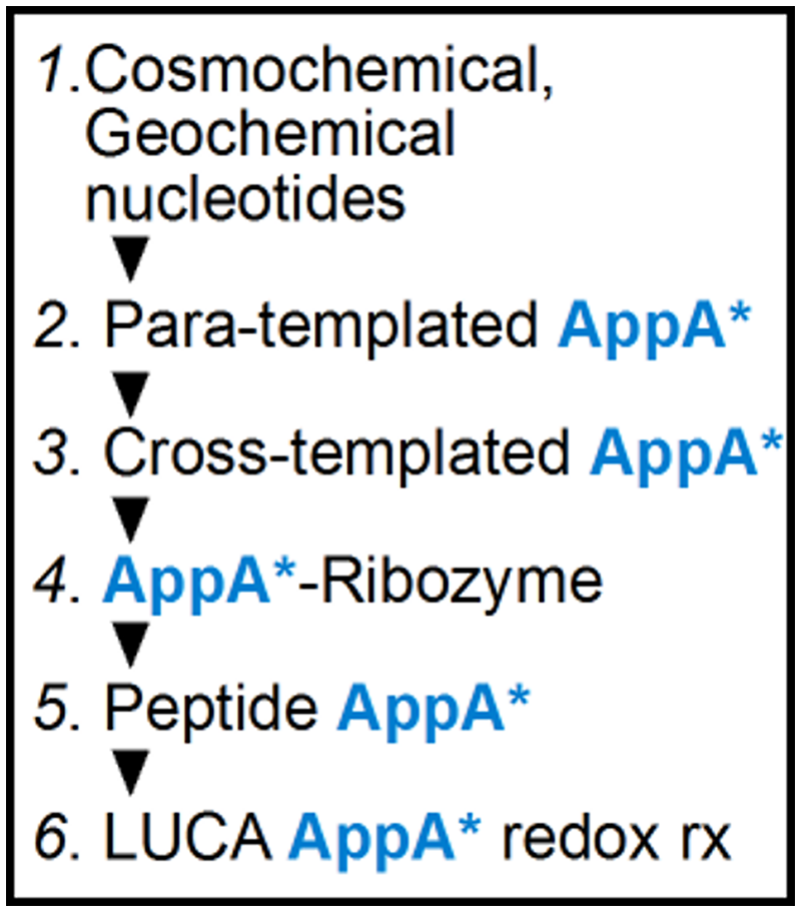
Evolutionary succession from nucleotides to a last universal common ancestor (LUCA). 1. Natural purine nucleotides (*1.*) initiate expression via localized para-templating of reactive dinucleotides (*2.*). When pyrimidine nucleotides later become available, the same product becomes more rapidly available from cross-templating (*3.*), still without need for complementary replication (Yarus 2017). Subsequent evolution of templated 5′–3′ RNA synthesis via base-pairing allows elaboration of an RNA world (*4.*) containing coenzyme-ribozymes (Jadhav and Yarus 2002). Later appearance of coded translation (Yarus 2001) creates modern peptide enzymes with bound RNA coenzymes (*5.*). These subsequently support a complex LUCA metabolism, whose redox reactions (*6.*) are particularly likely descendants of the original dinucleotides (*2*., see the Introduction). The coenzyme facsimile, AppA*, has been colored blue throughout the scheme to emphasize its continuous descent.

As might also be expected, NH_4_CN syntheses also yield other bases that pair as adenine, like 6-methyl A (Levy and Miller 1999) or 2,6-diaminopurine (Borquez et al. 2005). Thus, insofar as the nucleobases required for para-templating, both G and A as well as candidates for A* (Scheme 1) can come from a single localized purine synthesis event. This automatic unification of synthesis in one locale could make subsequent evolution of encoded gene expression (Yarus 2017) more probable. These advantages suggest that studies directed at natural synthesis of potential A*, that is, directed at unstable, metastable, or reactive nucleobase products, might be particularly interesting.

### Template and product backbones emerge from a single chemistry

Moreover, in para-templating, chemical formation of template and templated formation of product molecule backbones occur via a single kind of reaction. There is no requirement for mineral (or other) catalysis, as projected for cross-templating systems (Yarus 2017). We have argued that cross-templating is a likely primordial event because it requires only two nucleotides; one purine and a complementary pyrimidine (Yarus 2017). However, para-templating clearly has chemical requirements that are even more readily satisfied, and so might appear before cross-templating. Both kinds of templating can yield the same AppA-like product (Scheme 1), which somewhat resembles modern adenine-containing redox cofactors like NAD and FAD.

### A plausible path for evolution of coenzymes

We therefore suggest (Scheme 1) that para-templating preexisted, then gave way to cross-templating synthesis of the same coenzyme-like product, resembling AppA. This product was subsequently incorporated in aid of ribozyme reactivity, probably at the 5′ terminus, then ultimately exapted to extend the chemical repertoire of oligopeptides, as in Scheme 1.

Purine para-templating therefore eliminates previous reliance (Yarus 2017) on mineral assistance for the initial appearance of template—the para-templated pyrophosphate bond in both template GppG and product AppA is made in a solution reaction between 5′ phosphates. It is also clear why succession from initial para-templating to later cross-templating (*2.* and *3.* in Scheme 1) may have been favored. Para-templating of AppA is 24-fold slower than cross-templating on poly(U), so a pool under selection for product quantity could gain by switching to oligo(U) templates when they became available (Table 1).

### Para-templating probably allows evolution of coded expression

Significantly, previous logic (Yarus 2017) for the earliest appearance of encoded capabilities can still apply in a para-templating pool. Addition of GppG speeds overall synthesis of AppA (Fig. 2A,B), so para-templating may be selected in a sporadically fed pool if its coenzyme-like product has favorable activity. Selection of more productive pool chemistry (Yarus 2013) and consequent evolutionary progress via chance utility (Yarus 2016) may yield an inheritable phenotype via stimulatory pool effects and template RNA catalysis (Yarus 2017). We are investigating such an initially para-templated route to an inheritable chemical phenotype.

## MATERIALS AND METHODS

### Reactions

A typical reaction was held at 12°C, in 10 µL containing buffer and salts as in Table 1, plus 10 mM 2MeImpA, 10 mM pA (5′ AMP) with AMP marked by a trace amount of [^32^P]pA (Hartmann Analytic Gmbh). GppG was usually added to 10 or 12 mM. Samples of the reaction were held at −70°C until chromatography.

### Nucleotides

2MeImpA (adenosine 5′-phospho-2-methylimidazolide) was synthesized using the method of Joyce et al. (1984). Reactants of the form NppN (e.g., A5′pp5′A) were made following the method of Kanavarioti et al. (1991), subsequently HPLC-purified, lyophilized, and precipitated with ethanol overnight at −70°C at ∼70% yield. NppN are ultimately dissolved and stored in deionized H_2_O at −70°C. Identity was confirmed by mass spectrometry. Synthetic 3′–5′ pApA and pGpG (Thermo Fisher Scientific) were handled and stored as were other nucleotides.

### Chromatography

Samples were pipetted onto the origin on EMD-Millipore thin-layer PEI-cellulose F which had been eluted with deionized water and dried. Sheets are subsequently eluted at room temperature with 0.5 M LiCl (Randerath 1968). Chromatograms were dried and phosphorimaged (BioRad FX). [^32^P]AppA concentrations are calculated by multiplying the fraction of total cpm in the product spot by the known total concentration of AMP. Characteristic mobilities in Figure 1 for 2′–5′, 3′–5′, and 5′–5′ A dinucleotides were determined using characterized standards.

### Kinetics

When behavior of whole reactions was needed, the system's differential equations were numerically integrated using Berkeley Madonna v. 8.3.23.0 (https://www.berkeleymadonna.com). Post-kinetic processing of numerical data and graphic treatment took place in Microsoft Excel 2013. Models were fit to experimental data to determine k_chem_ (chemical synthesis rate, M^−1^ da^−1^), k_temp_ (templated synthesis rate, M^−2^ da^−1^), and k_deg_ (overall decay of activated nucleotides, da^−1^) listed in Table 1. The kinetic equations used were:

$$\begin{array}{c} d/dt(\;pA)=-k_{\mathrm{deg\_pA}}(\mathrm{pA})-k_{\mathrm{chem}}(\;\mathrm{pA})(\mathrm{ImpA})-k_{\mathrm{temp}}\left(\;\mathrm{pA})(\mathrm{ImpA})(\mathrm{GppG}\right)\;\\ d/dt(\mathrm{ImpA})=-k_{\deg}(\mathrm{ImpA})-k_{\mathrm{chem}}(\;\mathrm{pA})(\mathrm{Imp}A)-k_{\mathrm{temp}}\left(\;\mathrm{pA})(\mathrm{ImpA})(\mathrm{GppG}\right)\;\\ d/dt(\mathrm{GppG})=-k_{\mathrm{deg\_pG}}(\mathrm{GppG})\;\\ d/dt(\mathrm{AppA})=-k_{\mathrm{deg\_AppA}}(\mathrm{AppA})+k_{\mathrm{chem}}(\;\mathrm{pA})(\mathrm{ImpA})+k_{\mathrm{temp}}\left(\;\mathrm{pA})(\mathrm{ImpA})(\mathrm{GppG}\right)\; \end{array}$$

Rate constants used for decay of stable species, like *k*_deg_AppA_, are those listed in a prior paper (Yarus 2017). Full simulation code for the above system of equations is available on request. When full models were not required, initial least squares linear velocities from, e.g., four points in the first 0.33 da were used (compare Fig. 2B).
